# Fluid overload is associated with an increased risk for 90-day mortality in critically ill patients with renal replacement therapy: data from the prospective FINNAKI study

**DOI:** 10.1186/cc11682

**Published:** 2012-10-17

**Authors:** Suvi T Vaara, Anna-Maija Korhonen, Kirsi-Maija Kaukonen, Sara Nisula, Outi Inkinen, Sanna Hoppu, Jouko J Laurila, Leena Mildh, Matti Reinikainen, Vesa Lund, Ilkka Parviainen, Ville Pettilä

**Affiliations:** 1 Intensive Care Units, Division of Anaesthesia and Intensive Care Medicine, Department of Surgery, Helsinki University Central Hospital, Haartmaninkatu 4, 00290 Helsinki, Finland; 2 Department of Intensive Care, Turku University Hospital, Kiinamyllynkatu 4-8, 20520 Turku, Finland; 3 Department of Intensive Care and Emergency Medicine, Tampere University Hospital, Teiskontie 35, 33521 Tampere, Finland; 4 Division of Intensive Care, Department of Anaesthesiology, Oulu University Hospital, Kajaanintie 50, 90220 Oulu, Finland; 5 Department of Intensive Care, North Karelia Central Hospital, Tikkamäentie 16, 80210, Joensuu, Finland; 6 Department of Intensive Care, Satakunta Hospital District, Sairaalantie 3, 28500 Pori, Finland; 7 Division of Intensive Care, Kuopio University Hospital, Puijonlaaksontie 2, 70211 Kuopio, Finland; 8 Department of Clinical Sciences, University of Helsinki, Haartmaninkatu 4, 00290 Helsinki, Finland

## Abstract

**Introduction:**

Positive fluid balance has been associated with an increased risk for mortality in critically ill patients with acute kidney injury with or without renal replacement therapy (RRT). Data on fluid accumulation prior to RRT initiation and mortality are limited. We aimed to study the association between fluid accumulation at RRT initiation and 90-day mortality.

**Methods:**

We conducted a prospective, multicenter, observational cohort study in 17 Finnish intensive care units (ICUs) during a five-month period. We collected data on patient characteristics, RRT timing, and parameters at RRT initiation. We studied the association of parameters at RRT initiation, including fluid overload (defined as cumulative fluid accumulation > 10% of baseline weight) with 90-day mortality.

**Results:**

We included 296 RRT-treated critically ill patients. Of 283 patients with complete data on fluid balance, 76 (26.9%) patients had fluid overload. The median (interquartile range) time from ICU admission to RRT initiation was 14 (3.3 to 41.5) hours. The 90-day mortality rate of the whole cohort was 116 of 296 (39.2%; 95% confidence interval 38.6 to 39.8%). The crude 90-day mortality of patients with or without fluid overload was 45 of 76 (59.2%) vs. 65 of 207 (31.4%), *P *< 0.001. In logistic regression, fluid overload was associated with an increased risk for 90-day mortality (odds ratio 2.6) after adjusting for disease severity, time of RRT initiation, initial RRT modality, and sepsis. Of the 168 survivors with data on RRT use at 90 days, 34 (18.9%, 95% CI 13.2 to 24.6%) were still dependent on RRT.

**Conclusions:**

Patients with fluid overload at RRT initiation had twice as high crude 90-day mortality compared to those without. Fluid overload was associated with increased risk for 90-day mortality even after adjustments.

## Introduction

Four to eight percent of intensive care unit (ICU) patients receive renal replacement therapy (RRT) for acute kidney injury (AKI) [1-4]. The outcome of these severely ill patients remains poor with reported 90-day mortality rates varying between 45 and 74% [2,5-9]. The optimal timing of RRT initiation is unclear, although some studies suggest that early initiation might be beneficial [10,11]. The importance of fluid balance as a timing parameter has been highlighted [12,13]. An association of a greater degree of fluid accumulation at RRT initiation with higher mortality has been well documented in pediatric ICU patients [14,15]. Among adults, more positive mean daily fluid balance during ICU stay [16] or after RRT initiation [17] has been associated with increased mortality. Higher degree of fluid accumulation based on data on fluid balance from three days preceding nephrologist consultation [18] and on weight gain at RRT initiation in a retrospective cohort [19] has been associated with increased risk for death.

No studies presenting cumulative fluid balance from ICU admission to RRT initiation exist. Therefore, we aimed to evaluate the association of factors at RRT initiation and especially cumulative fluid accumulation with 90-day mortality in critically ill adults.

## Materials and methods

### Patients

We performed a prospective, observational cohort study, the FINNAKI study, from 1 September 2011 to 1 February 2012, in 17 Finnish ICUs. The referral areas of these ICUs cover 85% of the Finnish adult population. The Ethics Committee of the Department of Surgery, Hospital District of Helsinki and Uusimaa approved the study protocol and the use of deferred consent with a signed, informed consent from the patient or a proxy obtained as soon as possible. The Finnish National Institute of Health approved the collection of data from medical records of deceased patients if an informed consent could not be obtained.

**Figure 1:**
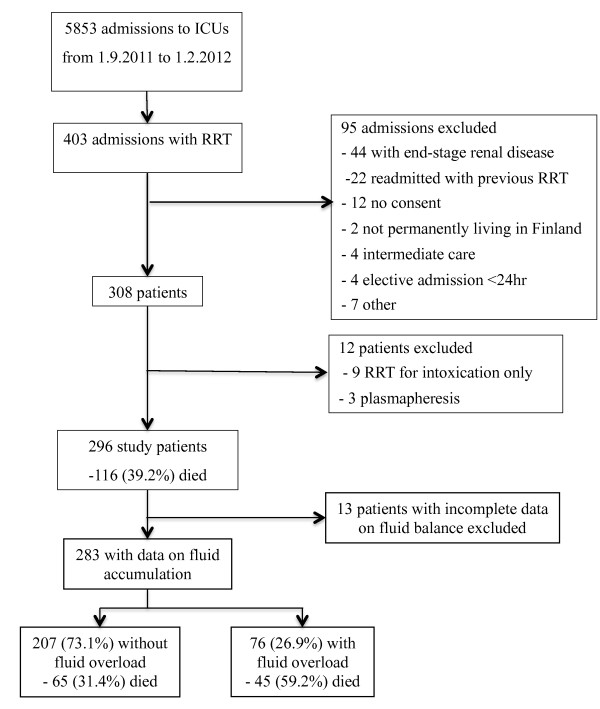
**Study flow chart with 90-day mortality rates**. *ICU, intensive care unit; RRT, renal replacement therapy.

We included all adult patients with either emergency ICU admission or elective post-operative ICU admission with an expected ICU stay of more than 24 hours. We excluded patients 1) with end-stage renal disease (ESRD) on chronic dialysis 2) who had already participated in the FINNAKI study and had received RRT during that previous ICU admission 3) not permanently living in Finland or having insufficient language skills for giving an informed consent, and 4) on intermediate care (Figure 1). Of patients receiving RRT, we excluded those who received RRT only for intoxication or plasmapheresis for non-renal indications. For analyses regarding fluid accumulation, we excluded patients with incomplete data on fluid balance. The decision to initiate RRT and the choice of RRT modality were based on the judgment of the treating clinician.

### Data collection

We collected physiological data, laboratory values, Sequential Organ Failure Assessment (SOFA) [20] and Simplified Acute Physiology Score (SAPS) II [21] scores, Acute Physiology and Chronic Health Evaluation (APACHE) III diagnoses, and patients' baseline weight with the help of the Finnish Intensive Care Consortium database using electronic patient records. We collected data on patients' chronic illnesses, baseline creatinine (latest creatinine value a week to a year prior to ICU admission), and treatment prior to ICU admission. The patients' daily fluid balance was calculated in the electronic patient records by summing the amount of fluids given (maintenance and resuscitation fluids, blood products, drug infusions, and enteral and parenteral nutrition), from which losses (urine output, bleeding, output from drains, rectal and nasogastric tube, and surrogate for evaporation (1000 mL for normothermic patients, and an addition per each Celsius degree of fever per hour)) were subtracted. We recorded data on patients' daily fluid balance from ICU admission to day five and obtained data on cumulative fluid balance of patients with RRT initiated after day five. We collected data on the given RRT treatment daily during the first five days and, thereafter, twice a week. We recorded the RRT replacement fluid flow and dilution mode, dialysis fluid flow, and the blood flow once daily. We calculated the CRRT dose (mL/kg/hr) according to previous equations [22] and considered treatment modality, blood flow, dilution mode, hematocrit, replacement and dialysis fluid flow, and the patient's weight. We recorded hospital mortality, length of ICU and hospital stay, and patients' need for RRT at 90 days from admission. We obtained patients' vital status 90 days from ICU admission from the Finnish Population Register Centre.

### Definitions

We calculated the total cumulative fluid balance from ICU admission to RRT initiation (including the day of RRT initiation) and defined the percentage of fluid accumulation by dividing the cumulative balance in liters by patient's baseline weight and multiplying by 100%. We then used the cutoff value of 10% of fluid accumulation as a definition of fluid overload [18,23]. We assessed presence of sepsis on admission and on days one to five according to the American College of Chest Physicians/Society of Critical Care Medicine definition [24]. We defined renal recovery as RRT independency at 90 days from ICU admission [25].

### Statistical methods

We report data as count and percentages or medians with interquartile range (IQR, 25^th ^to 75^th ^percentiles). We used Fisher's exact test to compare proportions and Mann-Whitney U-test to compare continuous data and calculated 95% confidence intervals (CI) for the main outcome. We studied factors associated with 90-day mortality with backwards logistic regression. We used stepwise elimination approach and a significance level of < 0.05 for entry and > 0.10 for removal. We entered the following variables: age, SAPS II score without age points, non-renal SOFA score on the day of RRT initiation, time from ICU admission to RRT initiation (days), initial RRT modality (continuous or intermittent), lactate (mmol/L), base excess (mmol/L), and plasma creatinine (umol/L) at RRT initiation, cumulative urine output on the day of RRT initiation, colloid use prior to RRT initiation (including data from ICU stay and 48 hours prior to ICU admission), presence of severe sepsis (yes/no) during the ICU admission, and fluid accumulation (%) at RRT initiation. We studied fluid overload (fluid accumulation > 10%) as a categorical variable in a separate model with all other covariates being the same as in the first model. We studied the potential interactions in separate models between degree of fluid accumulation (%) and 1) SAPS II score without age points, 2) day of RRT initiation, and 3) presence of severe sepsis 4) creatinine prior to RRT initiation and 5) urine output on the day of RRT initiation. We tested the goodness-of-fit with the Hosmer-Lemeshow test and calculated the area under the receiver operating characteristic curve (AUC) and correct classification rate. We examined the 90-day survival with Kaplan-Meier curves, and compared survival at 90 days between patients with and without fluid overload at RRT initiation with the log-rank test. We considered a two-sided *P *value lower than 0.05 as significant and made no corrections for multiple comparisons. In logistic regression, we set the significance level at 0.01. We performed the statistical analysis using the SPSS Statistics version 19.0 (SPSS Inc, Chicago, IL, USA).

## Results

We included 296 patients in the study (Figure 1). Source of admission and APACHE III diagnosis groups are presented in Table 1. The median (IQR) number of underlying co-morbidities was 1 (0 to 2), hypertension in 156 (53%), diabetes in 111 (37%), and coronary artery disease and/or peripheral vascular disease in 48 (16%) patients being the most common. Chronic kidney disease without the need for maintenance dialysis was reported in 47 (16%) patients. Patient characteristics, parameters at RRT initiation, and length of stay of all patients and a comparison between survivors and non-survivors are presented in Table 2.

**Table 1:** Source of admission and APACHE III admission diagnosis.

	All patients (*n *= 296)
**Source of admission**	

Emergency department	113 (38.2)
Operating room/recovery	91 (30.7)
Hospital ward	70 (23.6)
Another intensive care/high dependency unit	16 (5.4)
Other	6 (2.0)

**APACHE III admission diagnosis**	

**Non-operative**	**203 (68.6)**

Renal disease	51 (25.1)
Sepsis	38 (18.7)
Metabolic	32 (15.8)
Gastrointestinal disease	32 (15.8)
Cardiovascular	25 (12.3)
Other	25 (12.3)

**Operative**	**93 (31.4)**

Cardiovascular	51 (54.8)
Gastrointestinal disease	35 (37.6)
Other	7 (7.5)

Data presented as number (%). APACHE, Acute Physiology and Chronic Health Evaluation.

**Table 2:** Patient characteristics of all patients and compared between survivors and non-survivors.

	Data available	All *n *= 296	Survivors *n *= 180	Non-survivors *n *= 116	*P *value
Age (years)	296	64 [55-73]	62 [50-69]	70 [58-77]	< 0.001
Male gender	296	197 (66.6)	121 (67.2)	76 (65.5)	0.801
Baseline weight (kg)	296	80 [70-95]	85 [70-100]	76 [69-87]	0.001
Baseline creatinine (umol/L)	202	86 [70-116]	91 [71-117]	81 [68-114]	0.201
Emergency admission	292	271 (92.8)	164 (92.7)	107 (93.0)	0.900
Surgical admission	295	94 (31.9)	59 (33.0)	35 (30.2)	0.701
SAPS II score	296	51 [40-65]	46 [36-56]	63 [51-77]	< 0.001
SAPS II score without age points	296	40 [29-55]	36 [25-45]	51 [37-64]	< 0.001
Severe sepsis (during ICU stay)	296	142 (48.0)	73 (40.6)	69 (59.5)	0.002
Vasoactives (during ICU stay)	296	251 (84.8)	143 (79.4)	108 (93.1)	0.001
Mechanical ventilation (during ICU stay)	296	220 (74.3)	117 (65.0)	103 (88.8)	< 0.001

**Parameters at RRT initiation**					

SOFA score on RRT initiation day	290	10 [7-12]	9 [6-11]	12 [9-14]	< 0.001
Renal SOFA score on RRT initiation day	290	3 [2-3]	3 [1-4]	4 [2-4]	0.022
Cumulative balance on RRT initiation day (L)	283	3.8 [1.3-8.3]	3.1 [0.3-6.4]	6.2 [2.2-9.7]	< 0.001
Fluid accumulation on RRT initiation day (%)	283	5.1 [1.5-10.3]	3.6 [0.3-8.2]	8.0 [3.0-12.9]	< 0.001
Urine output mL/24 hr on RRT initiation day	278	325 [80-1191]	510 [157-1701]	150 [35-496]	< 0.001
Received diuretics prior to RRT initiation	296	202 (68.2)	117 (65.0)	85 (73.3)	0.160
Received colloids prior to RRT initiation	290	208 (71.7)	114 (64.4)	94 (83.2)	< 0.001
Amount of colloids received on ICU prior to RRT initiation (mL)	291	500 [0-1500]	500 [0-1250]	976 [138-2000]	0.001
Creatinine prior to RRT initiation (umol/L)	290	261 [168-427]	279 [169-506]	237 [164-336]	0.049
Lactate prior to RRT initiation (mmol/L)	276	2.4 [1.2-6.0]	1.8 [1.1-4.8]	3.8 [1.7-8.0]	< 0.001
pH prior to RRT initiation	291	7.29 [7.18-7.38]	7.31 [7.20-7.38]	7.25 [7.17-7.37]	0.072
Base excess prior to RRT initiation (mmol/L)	292	-8.8 [-13.6- -4.0]	-7.6 [-13.0- -3.4]	-10.0 [-13.9- -5.1]	0.046
Time from ICU admission to RRT initiation (hours)	290	14.0 [3.3-41.5]	10.9 [2.5-42.1]	19.2 [6.3-41.3]	0.039
CRRT as initial modality	296	215 (72.6)	121 (67.2)	94 (81.0)	0.011

**Length of stay (days)**					

ICU	296	5.5 [2.2-10.9]	6.0 [2.8-11.6]	4.0 [1.8-9.9]	0.039
Hospital	294	14.5 [6.0-27.3]	20.0 [11.0-32.0]	6.0 [2.0-18.0]	< 0.001

Continuous variables expressed as median [interquartile range]. Nominal variables expressed as number (%). ICU, intensive care unit; RRT, renal replacement therapy; SAPS, Simplified Acute Physiology Score; SOFA, Sequential Organ Failure Assessment.

Of 283 patients with complete data on fluid balance, 76 (26.9%) patients had fluid overload (Figure 1). Patients with fluid overload were more often admitted because of sepsis (25.0% vs. 8.2%, *P *< 0.001), while patients without fluid overload had renal disease as APACHE III admission diagnosis (22.7% vs. 2.6%, *P *< 0.001) more frequently. Other baseline characteristics and parameters at RRT initiation stratified according to fluid overload status are presented in Table 3. Patients with fluid overload at RRT initiation had a trend for a more positive daily fluid balance after RRT initiation (Additional file 1, Figure S1). No statistically significant differences in fluid removal with RRT existed between the groups (Additional file 2, Figure S2).

**Table 3:** Patient characteristics and outcome of patients with or without fluid overload.

	Data available	Fluid overload *n *= 76	No fluid overload *n *= 207	*P *value
Age (years)	283	64 [53-74]	64 [55-73]	0.934
Male gender	283	48 [63.2]	142 [68.6]	0.395
Baseline weight (kg)	283	75 [68-88]	83 [70-98]	0.004
Baseline creatinine (umol/L)	195	79 [68-99]	88 [70-126]	0.043
Emergency admission	279	67 (89.3)	192 (94.1)	0.192
Surgical admission	282	27 (35.5)	62 (30.1)	0.390
SAPS II score	283	55 [44-77]	50 [40-63]	0.025
SAPS II score without age points	283	44 [33-62]	40 [28-51]	0.016
Severe sepsis (during ICU stay)	283	49 (64.5)	88 (42.5)	0.001
Vasoactives (during ICU stay)	283	71 (93.4)	167 (80.7)	0.010
Mechanical ventilation (during ICU stay)	283	71 (93.4)	138 (66.7)	< 0.001

**Parameters at RRT initiation**				

SOFA score on RRT initiation day	277	11 [8-14]	10 [7-12]	0.002
Renal SOFA score on RRT initiation day	277	4 [3-4]	3 [2-4]	0.041
Cumulative balance on RRT initiation day (L)	283	10.5 [9.0-13.2]	2.4 [0.2-4.6]	< 0.001
Fluid accumulation on RRT initiation day (%)	283	13.1 [11.3-18.6]	3.0 [0.2-5.7]	< 0.001
Urine output mL/24 hr on RRT initiation day	265	164 [35-511]	429 [110-1531]	< 0.001
Received diuretics prior to RRT initiation	283	54 (71.1)	137 (66.2)	0.477
Received colloids prior to RRT initiation	278	69 (90.8)	131 (64.9)	< 0.001
Amount of colloids received on ICU prior to RRT initiation (mL)	281	1500 [564-2500]	400 [0-1000]	< 0.001
Creatinine prior to RRT initiation (umol/L)	279	206 [140-320]	281 [180-493]	0.001
Lactate prior to RRT initiation (mmol/L)	263	3.9 [1.8-7.2]	2.1 [1.2-6.0]	0.011
pH prior to RRT initiation	280	7.28 [7.18-7.38]	7.29 [7.18-7.38]	0.701
Base excess prior to RRT initiation (mmol/L)	281	-9.3 [-13.2- -4.3]	-8.6 [-14.4 - -4.0]	0.904
Time from ICU admission to RRT initiation (hours)	278	26.9 [10.7-43.7]	9.3 [2.5-35.7]	< 0.001
CRRT as initial modality	283	70 (92.1)	138 (66.7)	< 0.001

**Length of stay (days)**				

ICU	283	6.5 [2.2-15.0]	4.8 [2.1-9.9]	0.144
Hospital	281	12.5 [2.0-26.0]	15.0 [2.0-26.0]	0.037

**Crude mortality**				

Hospital	283	43 (56.6)	49 (23.7)	< 0.001
By day 90	283	45 (59.2)	65 (31.4)	< 0.001

**Dependent on RRT at 90 days (of survivors)**	162	3 (10.7)	31 (23.1)	0.202

Continuous variables expressed as median [interquartile range]. Nominal variables expressed as number (%). ICU, intensive care unit; SAPS, Simplified Acute Physiology Score; SOFA, Sequential Organ Failure Assessment.

### Renal replacement therapy

The median (IQR) number of reported indications for RRT was 3 (2 to 4). The most common indications were oliguria in 223 (78%), high creatinine in 196 (70%), acidosis in 181 (65%), and fluid accumulation in 116 (43%) patients. RRT was initiated on the first ICU treatment day in 124 (41.9%), on day two in 86 (29.1%), on day three in 37 (12.5%), and on day four or after in 49 (16.6%) patients. When patients were classified according to the day of RRT initiation, the degree of fluid accumulation increased until the day three (Additional file 3, Figure S3.) Of the 215 patients who initially received continuous RRT (CRRT), 111 (51.6%) received continuous veno-venous hemodialysis, 93 (43.3%) continuous veno-venous hemodiafiltration and 11 (5.1%) continuous veno-venous hemofiltration. The median (IQR) prescribed CRRT dose was 35.3 (31.2 to 40.6) mL/kg/hr (data from 205 of 215 patients). The median (IQR) daily duration of CRRT was 19.0 (9.3 to 24.0) hr (data from 680 CRRT treatment days), and CRRT dose adjusted for the treatment duration was 27.9 mL/kg/hr. The median (IQR) number of days patients who received CRRT (*n *= 230) was 3 (2 to 6) and number of intermittent RRT (*n *= 163) sessions 2 (1 to 4).

### Outcomes

**Figure 2:**
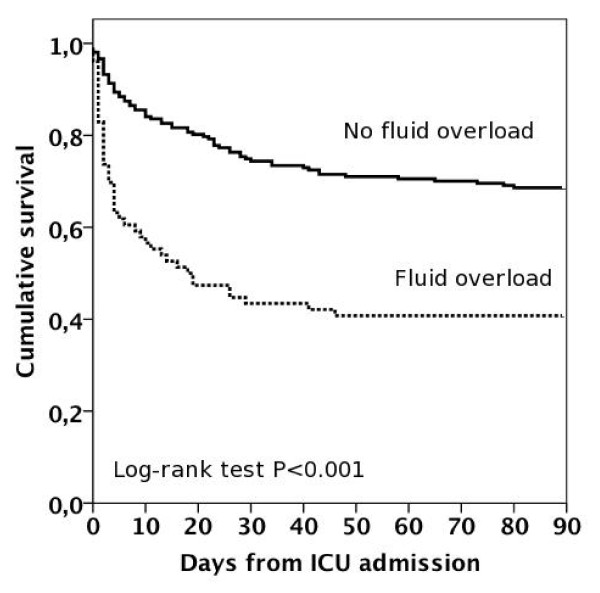
**Kaplan-Meier unadjusted survival curves for 90-day survival in patients with or without fluid overload**. *Number of patients with fluid overload 76 and without fluid overload 207.

**Figure 3:**
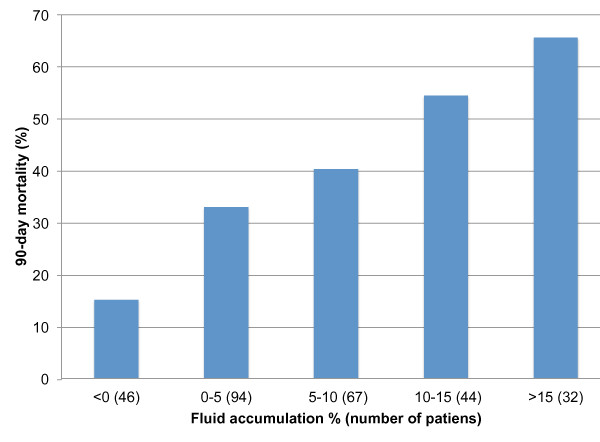
**Ninety-day mortality according to the percentage of fluid accumulation prior to renal replacement therapy initiation**. *Comparison across groups *P *< 0.001.

Of the 296 patients, 96 (32.4%; 95% CI 31.9 to 32.9%) died in hospital and 116 (39.2%; 95% CI 38.6 to 39.8%) before day 90. The SAPS II -based standardized mortality ratio (95% CI) was 0.64 (0.52 to 0.78). The hospital mortality of patients with or without fluid overload was 43 of 76 (56.6%) and 49 of 207 (23.7%), *P *< 0.001. The unadjusted 90-day survival of patients with fluid overload compared to those without is presented in Figure 2. The 90-day mortality rate increased as the degree of fluid accumulation increased (Figure 3). At 90 days, 34 of 168 (18.9%; 95% CI 13.2 to 24.6%) survivors (12 patients lost to follow-up) were dependent on RRT.

In logistic regression for 90-day mortality, no interactions between fluid accumulation percentage and 1) SAPS II score without age points, 2) time from ICU admission to RRT initiation, 3) presence of severe sepsis 4) creatinine prior to RRT initiation, or 5) urine output on the day of RRT initiation were present. The final model with fluid overload studied as categorical variable had Hosmer-Lemeshow chi-square 8.6 (*P *= 0.375), an AUC of 0.816 (95% CI 0.767 to 0.866), and correct classification rate of 74.4%. The results of the final model are presented in Table 4.

**Table 4:** Factors associated with 90-day mortality in logistic regression

	Odds ratio (95% confidence interval)	*P *value
SAPS II score without age points/point	1.048 (1.024-1.074)	< 0.001
Age (years)/year	1.046 (1.019-1.074)	0.001
Non-renal SOFA score^c^/point	1.218 (1.075-1.380)	0.002
Presence of fluid overload	2.626 (1.301-5.299)	0.007

^a^Only variables with a *P *< 0.01 are presented in the table. Non-significant variables: time from ICU admission to RRT initiation, initial RRT modality, urine output on the day of RRT initiation, colloid use prior to RRT initiation, presence of severe sepsis, lactate, base excess, and plasma creatinine at RRT initiation, ^b^number of patients included in the last step of the model 277, ^c ^on day of renal replacement therapy initiation. SAPS, Simplified Acute Physiology Score; SOFA, Sequential Organ Failure Assessment.

## Discussion

In this prospective, observational, nationwide cohort study in critically ill patients with RRT, the 90-day mortality rate was 39%. Median time from ICU admission to RRT initiation was 14 hours. Patients with fluid overload at RRT initiation had twice as high crude 90-day mortality compared to those without. Additionally, fluid overload was independently associated with an increased risk for 90-day mortality. Of 90-day survivors, 19% were dependent on RRT at 90 days.

We demonstrated an association between cumulative fluid overload at RRT initiation and increased risk for 90-day mortality, which remained significant after adjustment for disease severity, time of RRT initiation, initial RRT modality, patient's sepsis status, and several other parameters. The majority of non-survivors with fluid overload died in the hospital within a short time period, implying a severe course of disease. Fluid overload has been proposed as a biomarker of critical illness [26]. The mortality rate increased as the degree of fluid accumulation increased, which speaks for a potential dose-response relationship. RRT was initiated later on patients with fluid overload, and potentially, initiating RRT earlier could have altered their outcome.

In critically ill children, a strong association between fluid overload at CRRT initiation and mortality has been found [14,15]. In adults, a conservative fluid management strategy compared to a liberal strategy in patients with acute lung injury increased neither the incidence of AKI nor the need for RRT [27]. Among those with AKI, more positive fluid balance after AKI diagnosis was associated with higher adjusted mortality [28]. In a secondary analysis of the randomized evaluation of normal vs. augmented level (RENAL) study, positive mean daily fluid balance after RRT initiation was associated with worse outcome [6]. Payen *et al. *[16] demonstrated an association between higher mean daily fluid balance among AKI patients with and without RRT and 60-day mortality. Bagshaw *et al. *[29] found fluid accumulation over 5% of weight from 24 hours preceding RRT initiation to be associated with hospital mortality with an adjusted odds ratio (OR) of 2.31. Another study found fluid accumulation over 10% to be associated with 60-day mortality after adjusting for disease severity and RRT modality with an OR of 2.07 [18]. The calculations of cumulative balance were based on data from three days preceding nephrologist consultation, and neither the time point of the consultation nor the time between consultation and RRT initiation were specified [18,30]. A retrospective study also including patients treated outside ICUs [19] and a small prospective study [31] used weight at RRT initiation compared to baseline weight to determine fluid accumulation, and found that fluid accumulation was associated with an increased risk for mortality. A more positive fluid balance has been associated with increased risk for developing AKI after cardiovascular surgery [32] and a trend for increased need for RRT among septic shock patients with higher cumulative fluid balance has been noted [33]. Our results join the growing body of evidence showing an association between fluid overload and increased risk for mortality [14-19,29].

The 90-day mortality found in this study was lower compared to previous reports. In the RENAL study the 90-day mortality rate was 45% [6]. Randomization took place two days from ICU admission [6] and RRT was initiated later compared to our study. Furthermore, patients expected to die within 24 hours were excluded [6]. A prospective study also including patients treated outside ICUs reported a 90-day mortality rate of 48% [9]. In a large, retrospective cohort study, the 90-day mortality rate was 50%, with no disease severity score reported, but 85% of the patients initially received CRRT [5]. Several studies with higher disease severity scores have reported higher mortality rates from 56% [2,34] to 72% [35]. The early initiation of RRT in our study may be one plausible explanation for the lower 90-day mortality observed.

Hydroxyethyl starch solutions have been shown to increase mortality among patients with severe sepsis in the 6S trial [36]. In our study, 90% of patients with fluid accumulation received colloids prior to RRT initiation. Difference in colloid use was significant both between patients with or without fluid overload and survivors and non-survivors. However, colloid use did not remain significant in the logistic regression model for 90-day mortality. Moreover, the separation of the survival curves of patients with and without fluid overload occurred early after ICU admission, while the in the 6S trial the starch group separated from the Ringer's acetate group later [36].

The indications for RRT were in line with previous studies [6,37]. In few reports RRT has been initiated as early in terms of ICU treatment days [29,38], most studies reporting a median time from ICU admission to RRT initiation or randomization for RRT from two to seven days [6,7,39,40]. The proportion of patients initially receiving CRRT was higher than in a U.S. cohort with 56% [30] but corresponding to reports from Canada [29] and Taiwan [7] and slightly lower compared to a multinational study [38]. The initial CRRT dose adjusted for daily duration of CRRT was in line with the current recommendations [13].

Our study has some limitations. First, we were not able to record the fluid balance preceding ICU admission. However, in contrast to other studies [18,29] we recorded data on cumulative balance from ICU admission to RRT initiation day. Second, we could not relate the degree of fluid accumulation to physiologic parameters such as pulmonary capillary wedge pressure or stroke volume variation index. Third, regrettably, we did not collect data regarding the colloid type administered. Thus, we cannot separate to which extent the use of starch accounted for the worse outcome among patients with fluid overload. However, the use of any type of colloid did not remain significant in the logistic regression model. Fourth, the power of this study did not allow us to study the association between fluid accumulation and renal recovery. Finally, as this was an observational study, there may be factors that we did not measure that affected outcome, and the observed association does not imply causation. However, we reported data from a nationwide, non-selected patient cohort, with meticulously recorded prospective data about RRT timing.

## Conclusions

In this observational cohort study, the 90-day mortality of critically ill patients treated with RRT was 39%. Patients with fluid overload had twice as high 90-day mortality compared to those without. Fluid overload was associated with a markedly increased risk for 90-day mortality even after adjustments.

## Key messages

• AKI patients treated with RRT had a 90-day mortality of 39%.

• RRT was initiated early, a median of 14 hours after ICU admission.

• Patients with fluid overload (> 10% of weight) at RRT initiation had higher crude mortality compared to those without and had an increased risk for 90-day mortality after adjusting for disease severity, time of RRT initiation, RRT modality, and presence of severe sepsis.

• At 90 days, 19% of survivors were still dependent on RRT.

## Abbreviations

AKI: acute kidney injury; APACHE: Acute Physiology and Chronic Health Evaluation; AUC: area under the receiver operating characteristic curve; CI: confidence interval; CRRT: continuous renal replacement therapy; ICU: intensive care unit; IQR: interquartile range; OR: odds ratio; RRT: renal replacement therapy; SAPS: Simplified Acute Physiology Score; SOFA: Sequential Organ Failure Assessment.

## Competing interests

The authors declare that they have no competing interests.

## Authors' contributions

STV carried out the data analysis and drafted the manuscript. AMK, KMK and SN participated in designing the study. OI, SH, JL, LM, MR, VL and IP critically revised the manuscript. VP designed the study and helped to draft the manuscript. All authors participated in the data collection and read and approved the final manuscript.

## Supplementary Material

Additional file 1 **Figure S1: Median daily balance (mL) after renal replacement therapy initiation**. Click here for file

Additional file 2 **Figure S2: Median fluid removal (mL) with renal replacement therapy**. Click here for file

Additional file 3 **Figure S3: Percentage of fluid accumulation prior to RRT initiation according to RRT initiation day**. Click here for file
