# Supplementary figures and images for: Fluid overload is associated with an increased risk for 90-day mortality in critically ill patients with renal replacement therapy: data from the prospective FINNAKI study
Source: Crit Care. 2012 Oct 17;16(5):R197. doi: 10.1186/cc11682 (PMC3682299; doi:10.1186/cc11682)

Additional File Figure 1.

Median daily balance (mL) after renal replacement therapy initiation

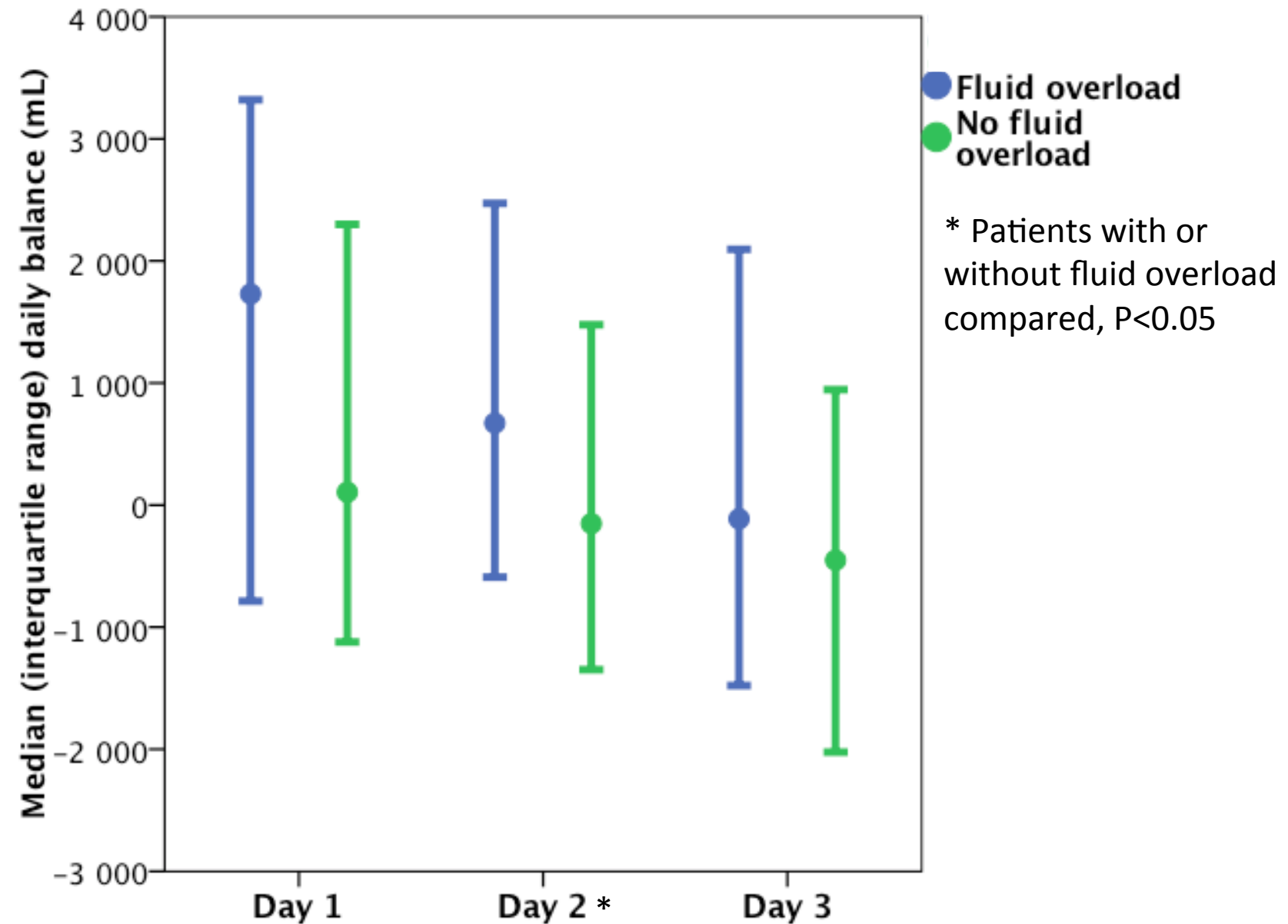

Supplement: Additional file 1 — Figure S1: Median daily balance (mL) after renal replacement therapy initiation. [file cc11682-S1.PDF]

Additional File Figure 2.

Median fluid removal (mL) with renal replacement therapy\*

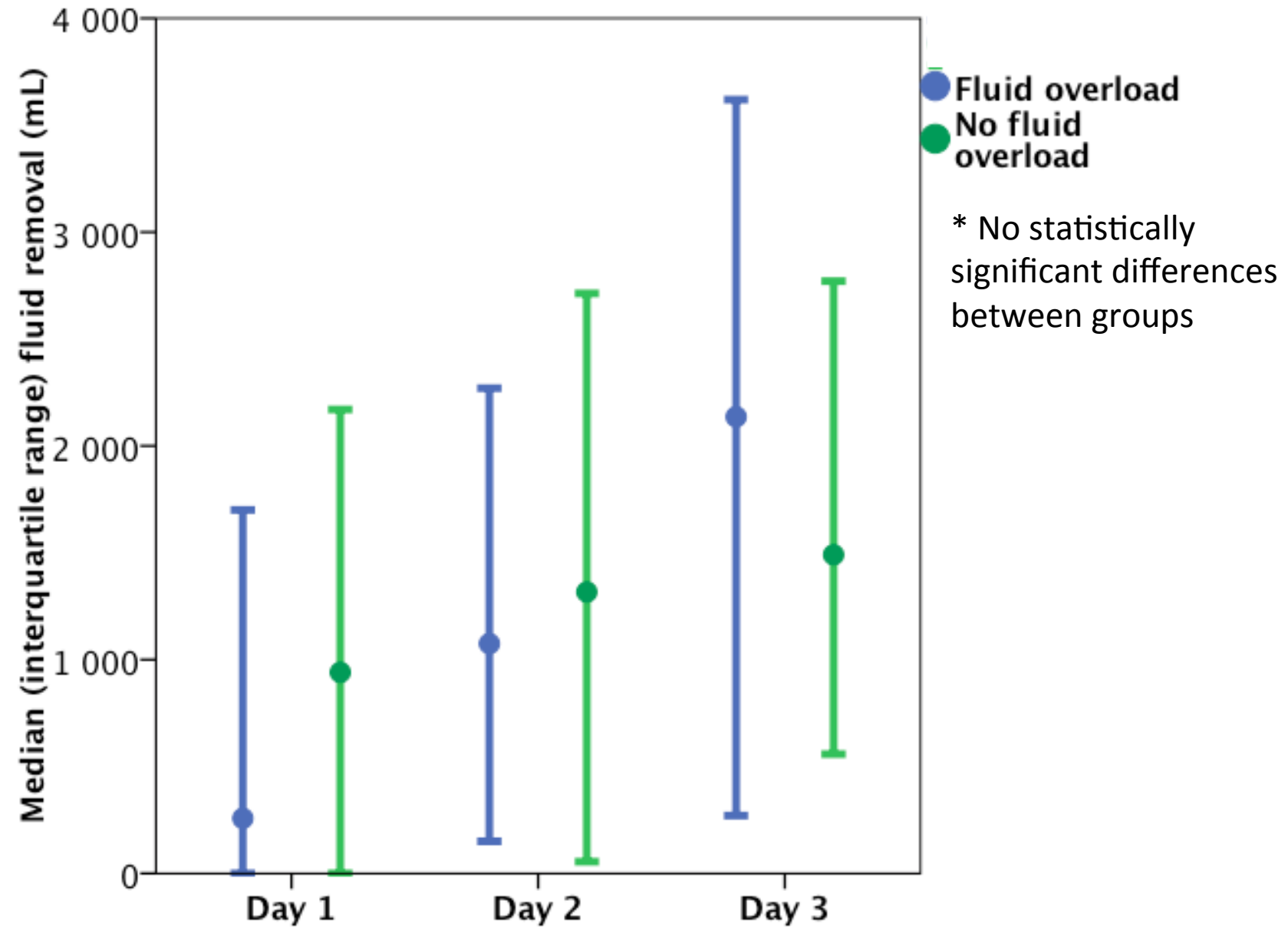

Supplement: Additional file 2 — Figure S2: Median fluid removal (mL) with renal replacement therapy. [file cc11682-S2.PDF]
