# Supplementary material for: Fluid overload is associated with an increased risk for 90-day mortality in critically ill patients with renal replacement therapy: data from the prospective FINNAKI study
Source: Crit Care. 2012 Oct 17;16(5):R197. doi: 10.1186/cc11682 (PMC3682299; doi:10.1186/cc11682)
Supplement: Additional file 3 — Figure S3: Percentage of fluid accumulation prior to RRT initiation according to RRT initiation day. [file cc11682-S3.PDF]

Additional File Figure 3.
Percentage of fluid accumulation prior to RRT initiation according to RRT initiation day.*

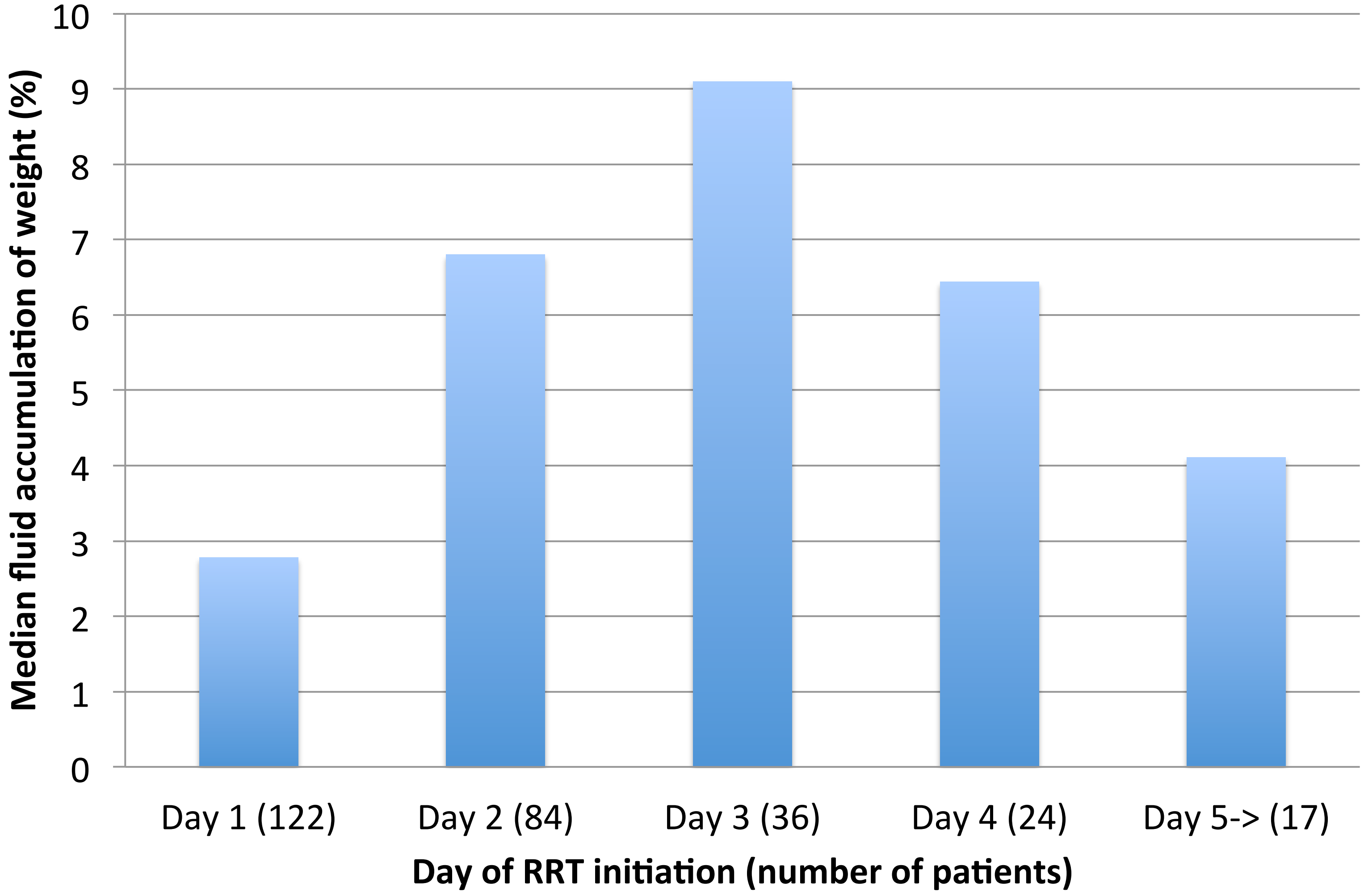

* Comparison across groups P=0.234, day 1 vs. day 2 or 3 P<0.001
